# Timing of mTOR activation affects tuberous sclerosis complex neuropathology in mouse models

**DOI:** 10.1242/dmm.012096

**Published:** 2013-06-05

**Authors:** Laura Magri, Manuela Cominelli, Marco Cambiaghi, Marco Cursi, Letizia Leocani, Fabio Minicucci, Pietro Luigi Poliani, Rossella Galli

**Affiliations:** 1 Neural Stem Cell Biology Unit, Division of Regenerative Medicine, Stem Cells and Gene Therapy, San Raffaele Scientific Institute, Via Olgettina 58, Milan 20132, Italy; 2 Department of Molecular and Translational Medicine, Pathology Unit, University of Brescia, Spedali Civili of Brescia, Brescia 25124, Italy; 3 Experimental Neurophysiology Unit, Institute of Experimental Neurology (INSPE), San Raffaele Scientific Institute, Milan 20132, Italy

## Abstract

Tuberous sclerosis complex (TSC) is a dominantly inherited disease with high penetrance and morbidity, and is caused by mutations in either of two genes, *TSC1* or *TSC2*. Most affected individuals display severe neurological manifestations – such as intractable epilepsy, mental retardation and autism – that are intimately associated with peculiar CNS lesions known as cortical tubers (CTs). The existence of a significant genotype-phenotype correlation in individuals bearing mutations in either *TSC1* or *TSC2* is highly controversial. Similar to observations in humans, mouse modeling has suggested that a more severe phenotype is associated with mutation in *Tsc2* rather than in *Tsc1*. However, in these mutant mice, deletion of either gene was achieved in differentiated astrocytes. Here, we report that loss of *Tsc1* expression in undifferentiated radial glia cells (RGCs) early during development yields the same phenotype detected upon deletion of *Tsc2* in the same cells. Indeed, the same aberrations in cortical cytoarchitecture, hippocampal disturbances and spontaneous epilepsy that have been detected in RGC-targeted *Tsc2* mutants were observed in RGC-targeted *Tsc1* mutant mice. Remarkably, thorough characterization of RGC-targeted *Tsc1* mutants also highlighted subventricular zone (SVZ) disturbances as well as STAT3-dependent and -independent developmental-stage-specific defects in the differentiation potential of *ex-vivo*-derived embryonic and postnatal neural stem cells (NSCs). As such, deletion of either *Tsc1* or *Tsc2* induces mostly overlapping phenotypic neuropathological features when performed early during neurogenesis, thus suggesting that the timing of mTOR activation is a key event in proper neural development.

## INTRODUCTION

Tuberous sclerosis complex (TSC) is a dominantly inherited disease with high penetrance and morbidity, and is caused by mutations in either *TSC1* or *TSC2*. TSC mostly affects children, who develop widespread lesions, known as hamartomas, in different organs, including the central nervous system (CNS). Hamartomas, which are considered the hallmark of the disease, comprise non-malignant cells that exhibit abnormal cell proliferation, aberrant cell differentiation and altered migratory ability. Typical CNS hamartomas include cortical tubers (CTs) – which are responsible for causing disabling neurological clinical features, such as infantile spasms (IS), epilepsy, mental retardation (MR) and autism – as well as asymptomatic subependymal nodules (SENs). The latter, through the evolution into subependymal giant cell astrocytomas (SEGAs), result in significant morbidity and mortality (Crino et al., 2006).

The existence of a significant genotype-phenotype correlation in individuals with TSC is still a matter of debate. Most individuals with *TSC2* mutations display a more severe neurological phenotype than those with mutations in *TSC1* (Dabora et al., 2001; Devlin et al., 2006; Jansen et al., 2008). However, only IS and epilepsy are strongly associated with *TSC2* mutations, whereas MR and neurocognitive impairment are linked to different types and location of *TSC1* and *TSC2* germline mutations, rather than to the specific gene in which the mutation occurred (van Eeghen et al., 2013). Similarly, the presence of SENs and SEGAs is not significantly associated with either gene mutation (Michelozzi et al., 2013), and variability in TSC symptoms has been reported in individuals with identical TSC mutations (Rok et al., 2005).

To reproduce experimentally TSC, different CNS-restricted conditional knockout murine models have been generated, by causing loss of either *Tsc1* or *Tsc2* in differentiating or differentiated neuronal cells (*Tsc1*^c/c^*/Syn*-Cre^+^ and *Tsc1*^c/c^*/CaMKII*-Cre^+^ mice) (Meikle et al., 2007; Ehninger et al., 2008) or in differentiated astrocytes [*Tsc1*^c/c^*/hGFAP*2.2kb (also known as *Tsc1*^c/c^*/hGFAP1*-Cre^+^) and *Tsc2*^c/c^*/hGFAP*2.2kb (also known as *Tsc2*^c/c^*/hGFAP1*-Cre^+^) mice] (Uhlmann et al., 2002a; Zeng et al., 2011). Given that CTs, SENs and SEGAs are believed to originate from a neural stem cell (NSC) undergoing abnormal differentiation, NSC-targeted mouse models of TSC have also been recently produced by deleting: (1) *Tsc2* in embryonic radial glial cells (RGCs) (*Tsc2*^c/−^*/hGFAP2*-Cre^+^ mice) at embryonic day 13.5 (E13.5) (Way et al., 2009), (2) *Tsc1* in *Emx1*-expressing embryonic dorsal telencephalic neuroepithelial progenitors (NEPs) at E9.5 (Magri et al., 2011; Carson et al., 2012), (3) *Tsc1* in embryonic E16.5 progenitors (Feliciano et al., 2011) and (4) *Tsc1* in postnatal SVZ NSCs (Zhou et al., 2011; Feliciano et al., 2012). Deletion of *Tsc1* or *Tsc2* at different developmental stages results in a gradient of phenotypes, with the most severe phenotypes being associated with mutations in early embryonic neural progenitors. As such, these same CNS-restricted TSC mouse models could be exploited to highlight potential genotype-phenotype correlations in TSC. As an example, conditional mice with *Tsc2* gene inactivation in differentiated astrocytes have been shown to display a more severe phenotype than those with *Tsc1* deletion (Zeng et al., 2011). Conversely, genetic inactivation of *Tsc1* and *Tsc2* in early embryonic neural progenitors such as NEPs (Magri et al., 2011) and RGCs (Way et al., 2009), respectively, resulted in very similar neocortical and hippocampal alterations, lamination defects, generation of enlarged cells, cell heterotopias, and epilepsy. Thus, as opposed to observations in differentiated astrocyte-targeted *Tsc1* or *Tsc2* mouse models, deletion of either *Tsc1* or *Tsc2* in distinct embryonic undifferentiated neural progenitors seems to result in overlapping phenotypes.

**TRANSLATIONAL IMPACT**

**Clinical issue**

Tuberous sclerosis complex (TSC) is a rare, dominantly inherited disorder associated with high penetrance and high morbidity. The disease, which is characterized by non-malignant tumor (hamartoma) development in multiple organs and severe neurological manifestations, is caused by mutations in either of two tumor suppressor genes, *TSC1* or *TSC2*. The existence of a significant genotype-phenotype correlation in individuals bearing mutations in *TSC1* or *TSC2* is a matter of debate. However, individuals with *TSC2* mutations have been shown to generally display a more severe neurological phenotype than those with mutations in *TSC1*. In agreement with this, knockout mouse models have provided evidence that a more severe neurological phenotype is associated with mutations in *Tsc2* rather than in *Tsc1*. In these earlier comparative studies, which implicate activation of the mTOR pathway as a contributory mechanism, genetic inactivation of *Tsc1* or *Tsc2* was limited to differentiated astrocytes. It has recently been shown that *Tsc2* loss in undifferentiated radial glial cells (RGCs; a type of neural stem cell) also recapitulates several neurological alterations associated with TSC. A similar investigation of the effect of *Tsc1* inactivation in undifferentiated RGCs on the mTOR pathway and TSC phenotypes has not been performed.

**Results**

In the present study, the authors address this issue by inducing *Tsc1* loss in undifferentiated RGCs, *in vivo* and *in vitro*. First, they generated and characterized in-depth a novel Cre-based conditional mouse model for targeted knockout of *Tsc1*. They report that deletion of *Tsc1* in hippocampal and cortical RGCs during early development results in neurological features that are reminiscent of TSC, some of which were detected in the corresponding *Tsc2* mutant mouse that was examined previously. Using this *Tsc1* conditional knockout mouse model, the group established long-term expanding postnatal NSC lines derived from the subventricular zone. In line with previous observations in other types of *Tsc1*-deficient NSC lines, mutant postnatal NSCs showed a robust decrease in self-renewal ability and defects in differentiation, which were ascribed to STAT3 hyperactivation. Importantly, inhibition of STAT3 rescued some of the differentiation defects observed *in vitro*, confirming the pivotal role of this pathway, downstream of mTOR activation, in regulating neurogenesis.

**Implications and future directions**

These results suggest that, contrary to earlier findings, loss of *Tsc1* results in neurological manifestations of TSC that are equivalent to those induced by loss of *Tsc2* in mutant mice. Moreover, mTOR activation was confirmed to play a crucial role in mediating the neurological abnormalities observed. The key difference between this work and earlier studies is that gene loss was assessed in NSCs rather than in differentiated cells. The data indicate that mTOR activation in neural cells can have different effects depending on the developmental stage at which it takes place, i.e. in immature or mature cells, and that genotype-phenotype correlation, at least in pre-clinical mouse models, might depend on the nature of the cells targeted by the mutation. Furthermore, the availability of developmental stage-specific NSCs provides a tool for testing different therapeutic approaches, as exemplified by STAT3 inhibition, for their effectiveness in rescuing the defects in neural stem cell neuropathology that underlie TSC and related disorders.

Because the undifferentiated progenitors NEPs and RGCs are in any case very different molecularly and functionally, to rigorously assess whether the phenotypes observed in *Tsc1* or *Tsc2* CNS-targeted mouse models depend on the distinct nature of the cells targeted by mutation (i.e. differentiated astrocytes versus early undifferentiated progenitors such as NEPs and RGCs), we decided to compare the effect of *Tsc1* loss in the very same progenitor cell type in which *Tsc2* deletion was previously performed, i.e. RGCs.

## RESULTS

### Targeted inactivation of *Tsc1* in RGCs causes shortened lifespan, megalencephaly, cortical alteration and spontaneous epilepsy

**Fig. 1.**
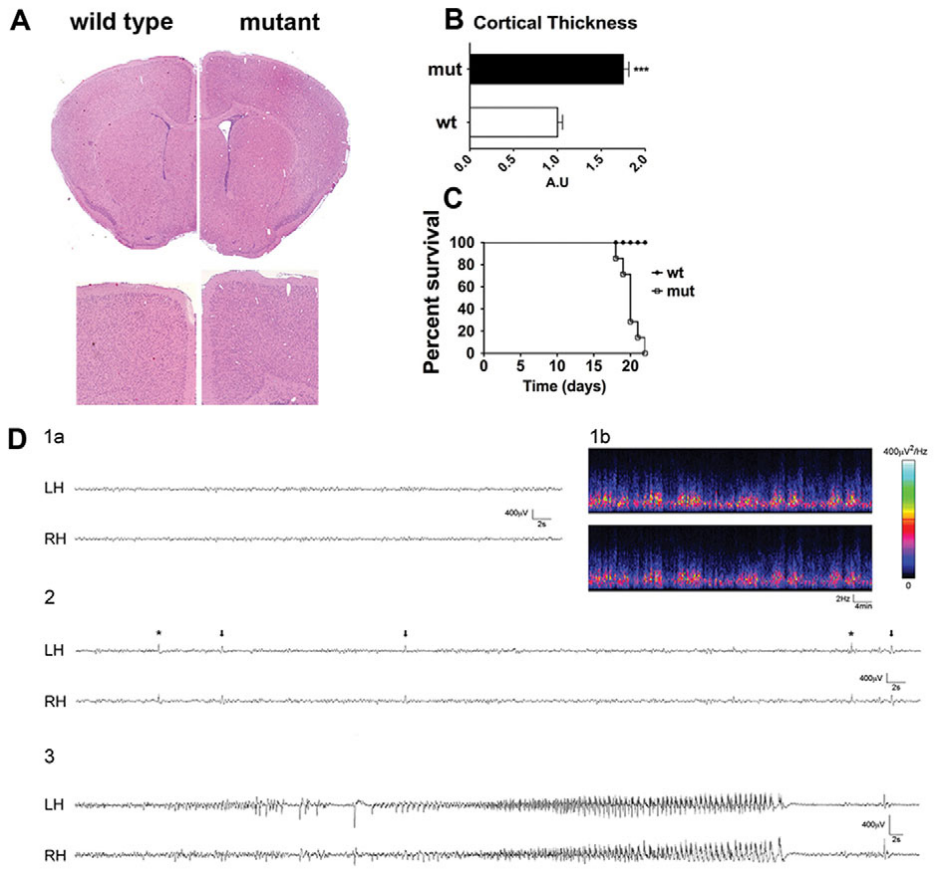
**Hyperactivation of the mTOR pathway in RGCs by conditional mutagenesis severely impairs CNS development and causes early lethality.** (A) H&E staining shows that the brains of *Tsc1*^c/−^*/hGFAP2*-*Cre*^+^ mutant mice are megalencephalic and characterized by increased cortical thickness as compared with control mice. Magnification 40× (upper panel), 100× (lower panel). (B) Quantification of the increase in cortical thickness. ***P<0.005. (C) Kaplan-Meier survival curve indicates that mutant mice have a shortened lifespan (*n*=12). Log-rank test, *P*<0.005. (D) EEG background activity in a representative mutant mouse (1a) recorded from left hemisphere (LH) and right hemisphere (RH). Density spectral arrays (DSA) corresponding to time-frequency analysis of the same animal (1b). (2) Epileptic abnormalities (sharp waves, arrows; spikes, asterisk) appear over background oscillatory activity; (3) a typical generalized cortical seizure is shown, characterized by a low-amplitude/high-frequency onset developing into a high-amplitude/low-frequency activity. When the seizure ends, a marked reduction in electrical activity is observed.

To explore the effect of *Tsc1* loss in RGCs, we interbred either *Tsc1*^c/c^ or *Tsc1*^c/−^ mice with *hGFAP2*-*Cre* mice, in which *Cre*-mediated recombination takes place in the hippocampal and cortical radial glia at E12 and at E13.5–E14, respectively (Zhuo et al., 2001). Both mutant mice were born in Mendelian ratios, and were indistinguishable from controls until postnatal day 10 (P10). Mutant brains at P15 were larger than in controls, mostly due to increased cortical thickness, and showed dilated lateral ventricles (Fig. 1A,B). Eventually, mutant mice died at 3 weeks of age (Fig. 1C).

Starting at P14, mutant mice showed a progressive decline in activity, acquired a humped posture and all developed spontaneous epileptic-like seizures. Video electroencephalographic (EEG) monitoring sessions beginning at P12 revealed that background activity was composed of sequences of oscillations of 1–4 Hz or 6–9 Hz (*n*=5 mice recorded; Fig. 1D, section 1a), which were also evident in density spectral array (DSA) plots (Fig. 1D, section 1b). In mutants, epileptic abnormalities were often superimposed on the background activity (Fig. 1D, section 2), especially after a seizure; a large spike frequently appeared just before or at the beginning of a seizure, without any behavioral correlate. High-amplitude sharp waves characterized seizure onset, followed by low-amplitude/high-frequency activity that progressively increased in amplitude and decreased in frequency. This trend evolved into a series of spike and wave complexes, ending with a marked decrease of EEG amplitude (Fig. 1D, section 3). Seizure length varied from 10 to 360 seconds, with a mean duration of 128±78 seconds. The mean frequency of seizures per hour was 0.14, with an average number of 3.5 seizures per day (supplementary material Table S1). Behaviorally, seizures started with head and truncus rhythmical jerks, followed by fore- and hind-limb myoclonic jerks. The Straub tail sign was also observed. Seizures ended with sudden immobility and progressive recovery of normal behavior. In some cases, animals died immediately after seizures.

Mutant mice in the present study did not show any overt sign of massive weight loss or cachexia, which, by contrast, has been observed in *Tsc1*^c/−^*/Emx1*-*Cre*^+^ mutant mice and contributed to their shortened lifespan (Magri et al., 2011). Indeed, hyperactivation of the mTOR pathway in selected hypothalamic nuclei that regulate food intake was observed in *Tsc1*^c/−^*/Emx1*-*Cre*^+^ mice, possibly due to leaky Cre activation. By contrast, although increased phosphorylation of ribosomal protein S6 at serine 235/236 (pS6_S235/6_) was observed in the hypothalamus of our mutant *Tsc1*^c/−^*/hGFAP2*-*Cre*^+^ mice, this was not ectopic and was retrieved in the same nuclei as in controls (supplementary material Fig. S1). As such, the short lifespan of *Tsc1*^c/−^*/hGFAP2*-*Cre*^+^ mutant mice was probably a consequence of the onset of status epilepticus, rather than of cachexia and wasting.

### Loss of Tsc1 in RGCs results in cortical alteration, enlarged cells, lamination defects, perturbed myelination and restricted mTORC1 activation in neurons

**Fig. 2.**
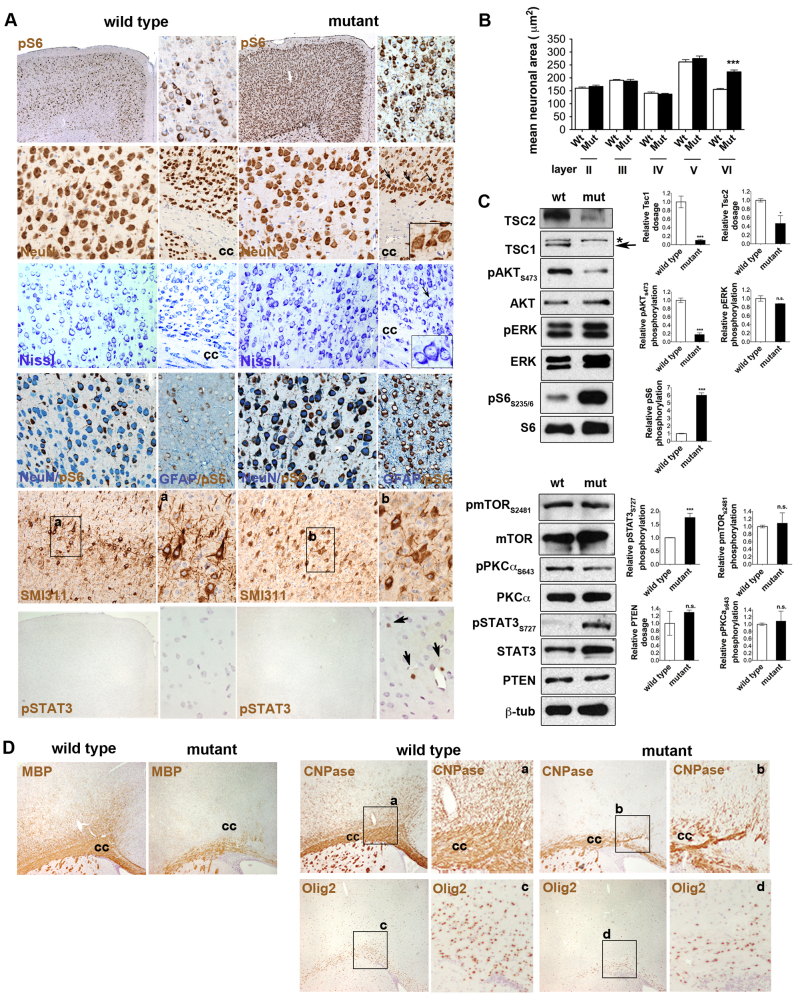
**Targeted inactivation of *Tsc1* in RGCs results in postnatal megalencephaly and cortical alterations.** (A) IHC on P15 control and mutant cortex for pS6_S235/6_, NeuN, Nissl, GFAP, Smi311 and pSTAT3. Increased cortical thickness and enhanced pS6 activation are observed in the cortex of P15 mutant mice. An ectopic pS6/NeuN-IR cell layer is detected in the mutant cortex (arrows and inset). No differences in cell size were seen throughout the control and mutant cortex, with the exception of cells located in the ectopic layers in the mutant cortex (Nissl staining). Most NeuN-IR neurons were also pS6-IR. By contrast, GFAP-IR cells do not hyperactivate pS6. Smi311-IR pyramidal neurons were profoundly disarranged in the mutant cortex (inset a and b). pSTAT3-IR cells were seen in P15 mutant cortex (arrows in high-magnification panel). Magnification 100×, insets 400×. (B) Quantification of cell size in the different layers of the P15 mutant and control cortex, as measured by unbiased automated cell area calculation, indicates enlarged cell size in the ectopic layer localized between layer VI and the CC in mutants. (C) Western blot on control and mutant cortex at P15. *: Tsc1 unspecific band. Arrow: Tsc1 specific band. Tsc1, Tsc2 and PTEN expression was normalized over β-tubulin, whereas pAKT_S473_, pERK, pS6_S235/236_, pmTOR_S2481_, pPKCα_S643_ and pSTAT3_S727_ activation was normalized over the corresponding total protein. Results are the average of the analysis of three samples per genotype. Densitometric analysis, error bars, s.e.m. (D) IHC on P15 control and mutant cortex for oligodendroglial markers such as MBP, CNPase (inset a and b) and Olig2 (inset c and d). Reduced myelination and a low number of Olig2-IR progenitors were detected in the mutant cortex compared with controls. Magnification 100×, inset 400×. cc, corpus callosum. *P<0.05; ***P<0.005.

Similar to *Tsc2*^c/−^*/hGFAP2*-*Cre*^+^ mice (Way et al., 2009), *Tsc1*^c/−^*/hGFAP2*-*Cre*^+^ P15 mutant brain showed clear signs of megalencephaly, lamination defects and microscopic cellular alterations (*n*=5 mice; Fig. 2A). Enhanced mTORC1 activation, as measured by pS6_S235/6_ hyperphosphorylation, and disorganization of NeuN-immunoreactive (IR) Nissl-positive neurons were observed in most layers of the cerebral cortex. In fact, an ectopic layer of NeuN-IR neurons was evident between layer VI and the corpus callosum (CC), and was uniquely characterized by increased cell soma size (Fig. 2B). Whereas most NeuN-IR cells in the *Tsc1*^c/−^*/hGFAP2*-*Cre*^+^ cortex activated pS6, GFAP-IR astrocytes, whose overall numbers were increased, did not (Fig. 2A). No differences in microglia activation were retrieved (supplementary material Fig. S1). Interestingly, SMI311-IR pyramidal neurons in layer V showed abnormal projections and disarranged neurites and axons. Notably, STAT3, which was previously identified as a crucial mediator of the premature differentiation occurring in *Tsc1*^c/−^*/Emx1*-*Cre*^+^ NSCs, was also hyperactivated in cells scattered through the *Tsc1*^c/−^*/hGFAP2-Cre*^+^ cortex *in vivo* (pSTAT3_Y705_, Fig. 2A, and pSTAT3_S727_, not shown).

In P15 mutant cortex, loss of Tsc1 and concurrent reduction in Tsc2 protein, which is in line with *TSC2* dependence on *TSC1* expression for stabilization of the *TSC1-TSC2* complex (Benvenuto et al., 2000), led to a marked increase in pS6_S235/6_ (Fig. 2C). Of note, pAKT_S473_ was strongly reduced, possibly due to the activation of an IRS1-dependent negative feedback and/or the loss of mTORC2 activity. However, differences in the activation of mTORC2, as measured by the downstream mediators pmTOR_S2481_ and pPKCα_S643_, were not detected (Fig. 2C). As opposed to *Tsc1*^c/−^*/Emx1*-*Cre*^+^ mutant cortex, no significant hyperactivation of pERK was retrieved in the *Tsc1*^c/−^/*hGFAP2*-*Cre*^+^ P15 cortex. In line with immunohistochemistry (IHC) findings, pSTAT3_S727_ was significantly overactivated in the *Tsc1*^c/−^*/hGFAP2*-*Cre*^+^ P15 mutant cortex. No difference in the expression of Pten was observed (Fig. 2C).

As in *Tsc2*^c/−^*/hGFAP2*-*Cre*^+^ mice, severe hypomyelination was observed in *Tsc1* mutants, as shown by a significant decrease in MBP- and CNPase-IR fibers (e.g. 0% and 43.9±4.3% MBP-negative hypomyelinated area/total area of the CC, in control and mutant brains, respectively; Student’s *t*-test, *P*<0.002) as well as in the number of Olig2-IR cells (39±7.0 and 15±7.0 cells/field in the CC in control and mutant brains, respectively; Student’s *t*-test, *P*<0.0001) (Fig. 2D).

Of note, loss of *Tsc1* in RGCs resulted in the same hippocampal enlargement and lamination alterations that were also evident in *Tsc2*^c/−^*/hGFAP2*-*Cre*^+^ brain (Way et al., 2009) (supplementary material Fig. S2). Specifically, the cornu ammonis (CA) 1 and 3 regions and the dentate gyrus (DG) of the mutant hippocampus showed aberrant splitting of the stratum pyramidale (sPy), ectopic localization of many NeuN-IR neurons and strong pS6 activation. Several ring heterotopias were present throughout the stratum lacunosum moleculare (SLM) and the hippocampal fissure. Doublecortin (DCX)-IR neuronal progenitors in the mutant subgranular zone (SGZ) of the DG displayed a highly extended neurite organization, mature morphology and increased size compared with controls (116±3 and 133±6 μm^2^, average area of control versus mutant cells, Student’s *t*-test, *P*<0.0001).

**Fig. 3.**
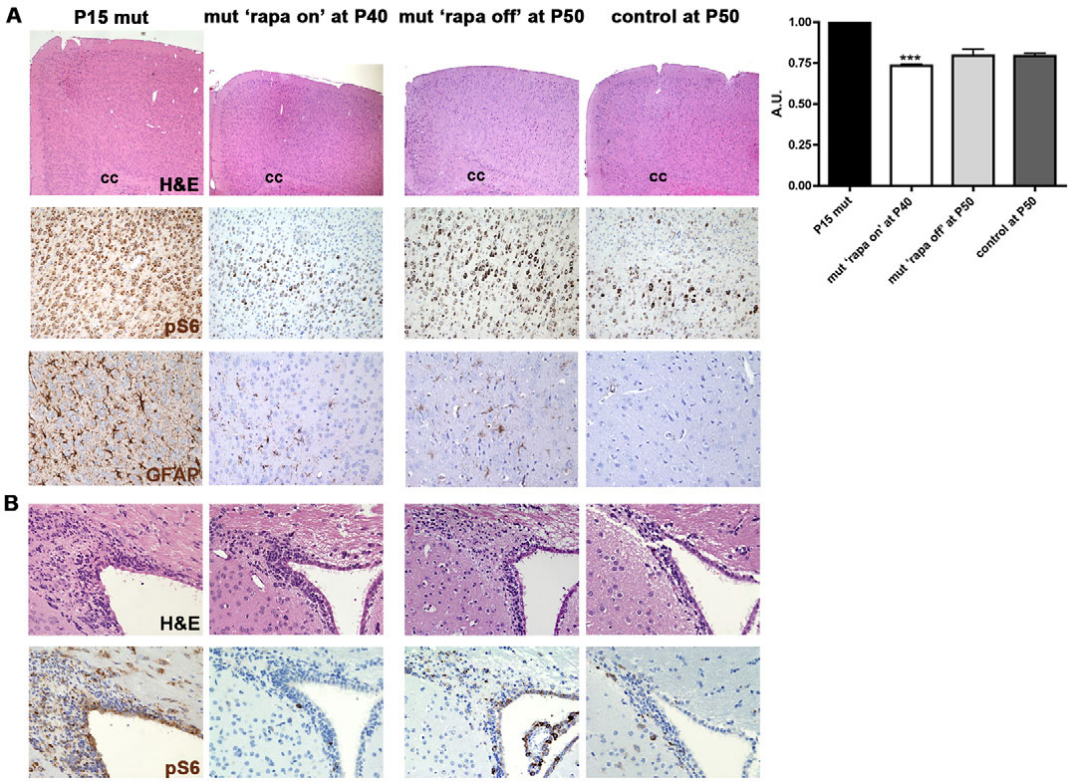
**Chronic rapamycin treatment reverts cortical and SVZ abnormalities.** (A) Chronic rapamycin treatment reduced pS6-IR cells and megalencephaly in P40 mutant ‘rapa on’ mice, probably through the reduction in cortical astrogliosis (GFAP-IR cells). Rapamycin discontinuation from P40 to P50 led to pS6 reactivation but did not affect the normalization of the cortical cytoarchitecture and the extent of reactive gliosis in mutant brains (mutant ‘rapa off’ mice) as compared with controls. cc, corpus callosum. Quantification of cortical thickness changes is shown on the right. ***P<0.005. (B) Chronic rapamycin treatment normalized SVZ alterations in P40 mutant ‘rapa on’ mice to control levels. Rapamycin discontinuation from P40 to P50 induced pS6 reactivation without promoting the reformation of SVZ aberrant structures in mutant brains (mutant ‘rapa off’ mice) as compared with controls.

To assess whether mTOR pharmacological inhibition can revert the epileptic and megalencephalic manifestations of the mutant phenotype, we delivered rapamycin from P8 to P40. All mutant mice treated by this regimen stopped developing seizures and were still alive at P40. Notably, both cortical thickness and S6 phosphorylation in rapamycin-treated mutant mice (mut ‘rapa on’) at P40 were identical to that of vehicle-treated wild-type mice (Fig. 3A). Because cortical neurogenesis does not take place postnatally, rapamycin-induced cortical thickness normalization might occur through other mechanisms, such as cortical neuropil remodeling. Indeed, rapamycin treatment induced a severe reduction in cortical reactive astrogliosis in the mutant cortex (Fig. 3A) (Magri et al., 2011). Within 10 days after rapamycin discontinuation, i.e. at P50, all mutant mice (mut ‘rapa off’) developed seizures and eventually died. Although pS6 hyperactivation was still seen in P50 ‘rapa off’ mutant cortices, cortical size did not increase back to the original mutant size, in line with the absence of *de novo* astrogliosis after rapamycin withdrawal (Fig. 3A).

### Loss of *Tsc1* in RGCs impairs the organization of the postnatal SVZ niche and induces the development of postnatal SEN-like lesions

**Fig. 4.**
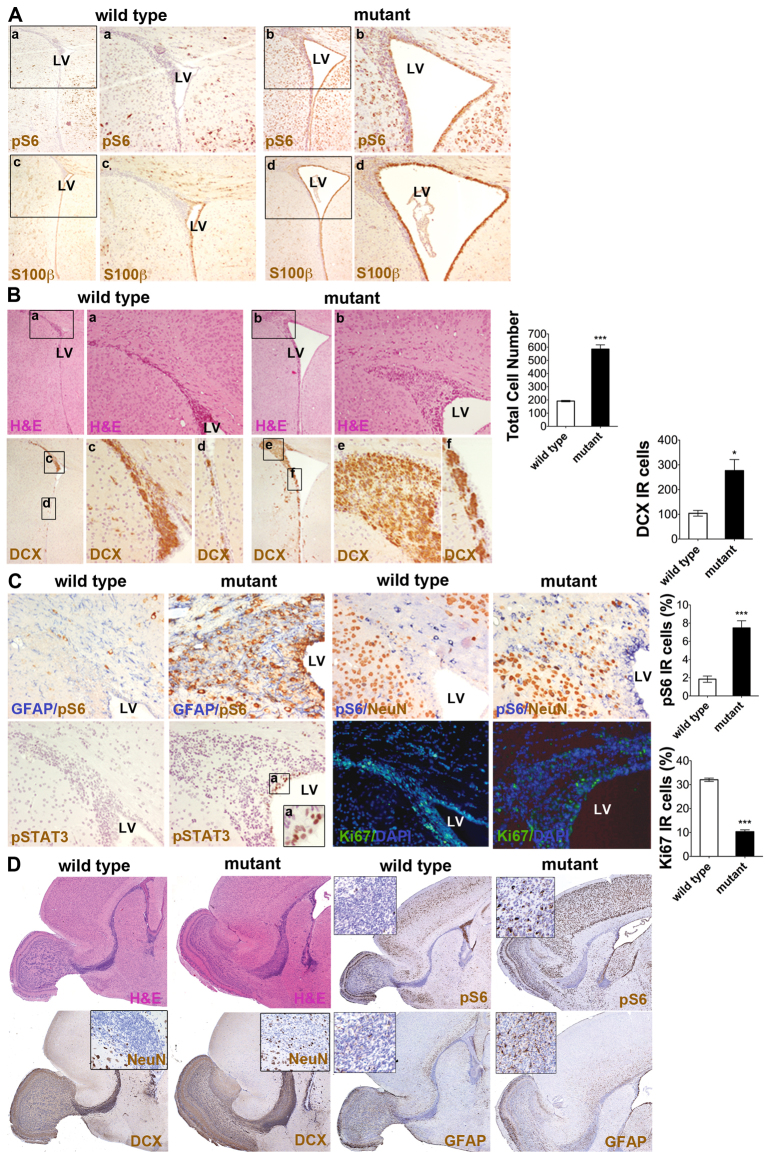
**Loss of *Tsc1* expression in RGCs severely impairs the organization of the postnatal SVZ niche.** (A) Enhanced dilatation of lateral ventricles (LVs) in mutant brains at P15. Enhanced pS6 activation (inset a and b) was seen along the whole lateral ventricles only in mutant brains. S100β expression (inset c and d) was similar in both controls and mutants. Magnification 40×, inset 100×. (B) H&E staining depicting the increased number of cells in the expanded SVZ in mutants (inset a and b). Increased numbers of type A progenitors immunoreactive for DCX in the SVZ expansion in mutants (inset c and e). Multilayered SVZ and DCX-IR cell clusters in the lateral wall of the mutant ventricle (inset d and f; coronal sections, 40×, 100×). (C) Higher frequency of GFAP-, pS6- and NeuN-IR cells is observed in the mutant SVZ than in controls (200×). GFAP-IR cells are pS6 negative. The mutant SVZ also contains some pSTAT3-IR cells, which are restricted to the lateral ventricle-verging side of the ventricular wall (inset a, 1000×). The frequency of Ki67-IR cells is decreased in the mutant SVZ (DAPI, blue; Ki67, green). (D) H&E, pS6, DCX, NeuN and GFAP staining highlights major defects in the organization of the mutant RMS at P15 (sagittal sections, 40×; insets, 200×). *P<0.05; ***P<0.005.

Although the analysis of SVZ neurogenesis was not reported for *Tsc2*^c/−^*/hGFAP2*-*Cre*^+^ mutant mice, we set out to assess whether mTORC1 hyperactivation in RGCs would result in SVZ alterations, as previously reported in *Tsc1*^c/−^*/Emx1*-*Cre*^+^ mice (Magri et al., 2011). At P15, the lateral ventricles of *Tsc1*^c/−^*/hGFAP2*-*Cre*^+^ mutant brains were abnormally enlarged (Fig. 1A; Fig. 4A). Indeed, as opposed to controls, S100β-IR ependymal cells in the mutant brain were all pS6-IR, suggesting that mTOR hyperactivation in these cells might affect their functionality, thus leading to ventricular enlargement.

Most interestingly, the same aberrantly expanded SVZ region previously detected in postnatal *Tsc1*^c/−^*/Emx1*-*Cre*^+^ mice was observed also in *Tsc1*^c/−^*/hGFAP2*-*Cre*^+^ mutant brain (Fig. 4B). This expansion was reminiscent of typical TSC-associated neuroglial SENs because it contained both DCX-IR neuroblasts, also organized as small heterotopic clusters along the entire lateral wall of the SVZ (Fig. 4B), and GFAP-IR astrocytes (Fig. 4C). Remarkably, aberrant expression of the neuronal marker NeuN was seen in the mutant SVZ, suggesting premature differentiation of mutant SVZ neuroblasts, which normally differentiate into mature neurons only after reaching their final destination, i.e. the olfactory bulbs (OBs) (Petreanu and Alvarez-Buylla, 2002). Similar to the mutant cortex, GFAP-IR cells in the SVZ did not activate pS6 (Fig. 4C). Notably, pSTAT3-IR cells were seen along the dorsal wall of the SVZ in close contact with the lateral ventricles (Fig. 4C). In agreement with the benign nature of human SENs, the percentage of Ki67-IR cells in the aberrant mutant SVZ expansion at P15 was lower than in controls (32.1±0.7% and 10.3±0.7% in P15 control and mutant SVZ, respectively; Fig. 4C).

In line with SVZ alterations, loss of *Tsc1* expression in RGCs led to defects in the organization of the rostral migratory stream (RMS). Indeed, the mutant RMS was thickened and contained many pS6-IR cells, multiple layers of DCX-IR cells and increased numbers of NeuN-IR and GFAP-IR cells (Fig. 4D), compared with controls. Accordingly, the anatomical targets of neuroblast migration along the RMS, i.e. the OBs, were morphologically abnormal (Fig. 4D).

Again, both the ventricular enlargement and the abnormal SVZ expansion were reverted in rapamycin-treated mutant mice, and did not reoccur upon rapamycin suspension, in spite of pS6 reactivation (Fig. 3B) (Magri et al., 2011).

### Severe alterations in cortical development and SVZ organization are detected at E18.5 and maintained postnatally

**Fig. 5.**
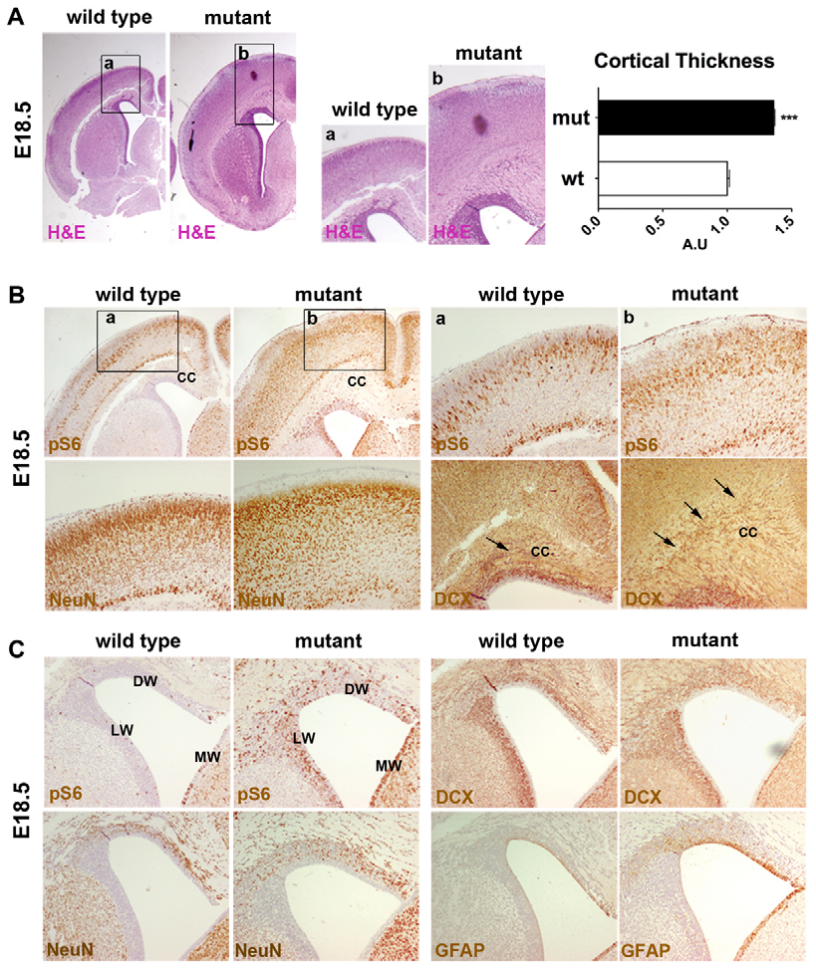
**Defects in the organization of the mutant cortex and SVZ are already detected at late embryonic developmental stages.** (A) Increased cortical thickness in the mutant brain is observed at E18.5 (coronal sections, 40× and inset a and b, 200×). ***P<0.005. Error bars, s.e.m. (B) Enhanced activation of pS6 in the mutant cortex at E18.5 (40× and inset a and b, 200×). Lamination defects in the mutant cortex are highlighted by NeuN and DCX staining (magnification 200×). DCX-IR cells massively infiltrate the prospective CC (arrows). (C) Higher number of pS6-, DCX-, NeuN- and GFAP-IR cells in the mutant SVZ at E18.5 (100×). DW, dorsal wall of the ventricle; LW, lateral wall of the ventricle; MW, medial wall of the ventricle.

**Fig. 6.**
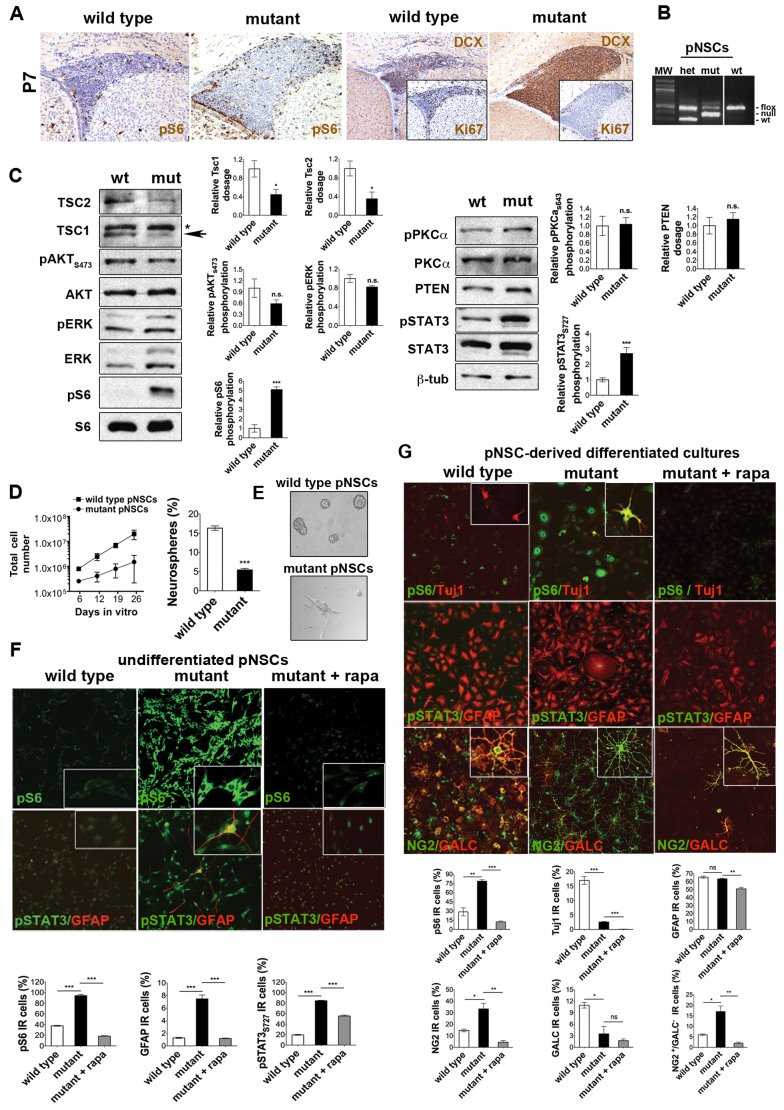
**Activation of the mTOR pathway in P7 postnatal NSCs finely regulates their self-renewal ability and their differentiation**. (A) Higher frequency of pS6- and DCX-IR cells in the aberrantly expanded mutant SVZ at P7. A lower frequency of Ki67-IR cells was detected in mutant SVZ (200×). (B) PCR analysis on genomic DNA from single mouse-derived control (wt) and mutant (mut) postnatal (p)NSCs, showing full recombination of the floxable allele. (C) Western blot on P7 control and mutant pNSCs. *: TSC1 unspecific band. Arrow: TSC1 specific band. TSC1, TSC2 and PTEN expression was normalized over β-tubulin, whereas pAKT_S473_, pERK, pS6_S235/236_, pmTOR_S2481_, pPKCα_S643_ and pSTAT3_S727_ activation were normalized over the corresponding total protein. Results are the average of three pNSC lines per genotype. Densitometric analysis, error bars, s.e.m. (D) Long-term growth curve and clonal efficiency of control and mutant pNSCs, indicating reduced self-renewal in mutant pNSCs. (E) Phase-contrast microphotographs of control and mutant pNSCs. Magnification 200×. (F) Increased S6 phosphorylation, premature astrocytic differentiation and pSTAT3 phosphorylation were detected in mutant pNSCs and were reverted by rapamycin. Immunofluorescence showing nuclear localization of pSTAT3_S727_ in ectopic GFAP-IR cells in undifferentiated mutant pNSC cultures. Magnification 200×. (G) Increased S6 phosphorylation and aberrant differentiation of pNSCs into GFAP-IR astrocytes after FBS addition was fully rescued by rapamycin treatment. Defective oligodendrogenesis was restored by rapamycin in terms of number of NG2 oligodendrocyte progenitors, whereas impaired maturation of Tuj1-IR neurons was not (300×). Magnification 200×. Error bars, s.e.m. *P<0.05; **P<0.01; ***P<0.005.

As opposed to *Tsc2*^c/−^*/hGFAP2*-*Cre*^+^ mice, increased cortical thickness and SVZ disturbances were already observed in *Tsc1*^c/−^*/hGFAP2*-*Cre*^+^ brains at E18.5 (Fig. 5A) and maintained at P7 (not shown and Fig. 6A). Similar to P15 mutant brain, pS6 activation at E18.5 was seen throughout all the cortical layers (Fig. 5B). The CC in the mutant brain was also highly enlarged as compared with controls. Interestingly, NeuN staining highlighted disorganized cortical lamination in mutants, with evident loss of neuronal alignment in the upper cortical layers as well as increased intercellular matrix and enlarged neuropil in the lower cortical layers (Fig. 5B). Ectopic invasion of the prospective CC by DCX-IR cells was specifically detected in the mutant cortex.

As opposed to *Tsc1*^c/−^*/Emx1*-*Cre*^+^ mice, the SVZ expanded region in *Tsc1*^c/−^*/hGFAP2*-*Cre*^+^ mice at late embryonic and/or perinatal stages did not protrude into the ventricle. On the contrary, the *Tsc1*^c/−^*/hGFAP2*-*Cre*^+^ mutant SVZ consisted of thickened multilayered cellular areas that lined the whole ventricle (Fig. 5C). A high number of pS6-IR cells, DCX-IR neuroblasts and NeuN-IR neurons was observed all around the mutant ventricle, whereas increased numbers of GFAP-IR cells were found in the dorsal and medial walls.

### Activation of mTORC1 in postnatal NSCs reduces their self-renewal, induces premature astroglial differentiation, and inhibits neuronal and oligodendroglial cell maturation

Whereas at E18.5 the frequency of Ki67-IR cells in control and mutant SVZ was similar (57.1±2.1% and 57.5±2.5%, respectively), the frequency of Ki67-IR in the mutant SVZ postnatally was significantly lower than in controls (54.0±1.0% and 13.0±1.0% in P7 control and mutants, respectively; Fig. 6A for P7 and Fig. 4C for P15). Thus, a progressive decline in the proliferation of mutant SVZ progenitors was detected throughout postnatal development.

To assess the role of mTOR pathway hyperactivation in postnatal SVZ NSCs *ex vivo*, we established long-term expanding NSC lines at P7. At this postnatal stage, both mutant cortex (data not shown) and SVZ displayed the same alterations as detected at P15, including enhanced pS6 activation, reduced proliferation and hypomyelination (Fig. 6A and not shown). As opposed to dorsally located *Tsc1*^c/−^*/Emx1*-*Cre*^+^ postnatal SVZ NSCs, which *in vitro* were taken over by non-recombined laterally and medially located postnatal SVZ NSCs, *Tsc1*^c/−^*/hGFAP2*-*Cre*^+^ postnatal SVZ NSCs showed almost complete *Cre*-mediated *Tsc1* loss at the genomic level, indicating that, in these mutants, recombination was taking place in all NSCs lining the walls of the lateral ventricles, thus allowing the establishment of efficiently recombined postnatal NSC lines (Fig. 6B).

Tsc1 and Tsc2 expression was significantly reduced and S6 phosphorylation highly increased in all mutant cultures (*n*=3 different pairs of postnatal NSC lines) (western blot, Fig. 6C). Phosphorylation of STAT3_Ser727_ was higher in mutant postnatal NSCs than in control, whereas pAKT, pERK, PTEN and pPKCα levels did not change. Interestingly, compared with controls, a significant decrease in long-term self-renewal and in clonal efficiency was observed in mutant postnatal NSCs (Fig. 6D), which, even in the presence of EGF and FGF2, acquired the appearance of highly differentiated cells (Fig. 6E). Indeed, undifferentiated mutant cultures, which were mostly pS6- and pSTAT3-IR, comprised many GFAP-IR astrocyte-like cells that displayed an aberrant morphology (Fig. 6F). In line with the role of STAT3 in promoting GFAP expression by binding to its promoter, most GFAP-IR cells were also pSTAT3-IR (Fig. 6F). Interestingly, mTORC1 inhibition by rapamycin reduced pS6 activation and reverted the premature glial differentiation in mutant postnatal NSCs to control levels (Fig. 6F).

To assess whether the mTOR pathway might also regulate the multilineage differentiation potential of postnatal NSCs and the maturation of their progeny, control and mutant postnatal NSCs were induced to differentiate and to mature for up to 8 days *in vitro* (Fig. 6G). After 3 days in the presence of the sole FGF2, a condition that drives the commitment of postnatal NSCs into bipotent/unipotent progenitors, all mutant postnatal NSC-derived progenitor cells activated pS6, but no difference was observed in terms of neuronal lineage commitment as compared with controls (not shown). By contrast, under maturation-promoting culture conditions, mutant postnatal NSC-derived cultures, which still had activated pS6 in most cells, were almost completely devoid of Tuj1-IR neuronal cells but comprised a normal number of GFAP-IR astrocytes, although the morphology of these cells was abnormal. Most notably, in agreement with the hypomyelination detected in the mutant CC *in vivo* at P15 (Fig. 2D), the number of GalC-IR oligodendrocytes in mutant postnatal NSC-derived differentiated cultures was strongly reduced compared with controls, a phenomenon that was not observed in embryonic NSCs isolated at E16.5 from *Tsc1*^c/−^*/Emx1*-*Cre*^+^ mice. Depletion of GalC-IR oligodendrocytes was probably due to a defect in the maturation of NG2-IR precursors into mature oligodendrocytes, as suggested by the increased frequency of NG2^pos^/GalC^neg^ cells in mutant cultures (Fig. 6G).

Once more, treatment of postnatal NSC-derived progeny with rapamycin led to normalization in the morphology of aberrant GFAP-IR cells and decreased the number of NG2^pos^ cells to control levels (Fig. 6G). However, rapamycin was ineffective in rescuing the loss in Tuj1-IR neurons and GalC-IR oligodendrocytes. Overall, mTOR hyperactivation in postnatal neural progenitors promoted early astroglial commitment and differentiation, followed by dysfunctional neuronal and oligodendroglial maturation.

To understand whether the impaired oligodendroglial differentiation observed exclusively in *Tsc1*^c/−^*/hGFAP2*-*Cre*^+^ postnatal NSCs was related to the stage of development at which the NSC cultures were established (i.e. postnatal versus embryonic) or to the progenitor cell type in which *Tsc1* deletion originally took place (i.e. RGCs versus NEPs), we established cortical embryonic NSCs from *Tsc1*^c/−^*/hGFAP2*-*Cre*^+^ mice at E16.5, i.e. the same time point at which we previously isolated embryonic NSCs from *Tsc1*^c/−^*/Emx1*-*Cre*^+^ mice (supplementary material Fig. S3). When maintained under proliferative culture conditions, *Tsc1*^c/−^*/hGFAP2-Cre*^+^ embryonic NSC lines showed a significant decrease in long-term self-renewal and in clonal efficiency (supplementary material Fig. S3A), and acquired the appearance of differentiated cells (supplementary material Fig. S3B). Most mutant embryonic NSC lines showed complete *Tsc1* loss (supplementary material Fig. S3C). Again, mutant embryonic NSCs, which were all pS6-IR, comprised many abnormal GFAP-IR cells and hyperactivated the STAT3 pathway (supplementary material Fig. S3D). After 3 days in FGF2, in contrast with postnatal NSCs, mutant pS6-IR embryonic NSC-derived progenitors gave rise to a higher number of Tuj1- and GFAP-IR cells than did controls, confirming premature differentiation (supplementary material Fig. S3E). Under maturation-promoting culture conditions, mutant cultures showed a higher frequency of pS6-, pSTAT3-, Tuj1- and GFAP-IR cells, again indicating both mTORC1-STAT3 pathway activation and enhanced neuronal and astroglial differentiation. Most notably, similar to embryonic NSCs from *Tsc1*^c/−^*/Emx1*-*Cre*^+^ mice, RGC-targeted *Tsc1* mutant embryonic NSCs comprised the same frequency of GalC-IR oligodendrocytes as control cultures, with only a slight increase in the numbers of NG2^pos^/GalC^neg^-IR progenitor cells (supplementary material Fig. S3F). Thus, impaired oligodendrogenesis is a unique feature of postnatal NSCs and does not relate to the type of embryonic neural progenitors (NEPs vs RGCs) targeted by mutation.

### Premature astroglial differentiation and impaired neuronal maturation of postnatal NSCs are STAT3-dependent, whereas defective oligodendrogenesis is STAT3-independent

**Fig. 7.**
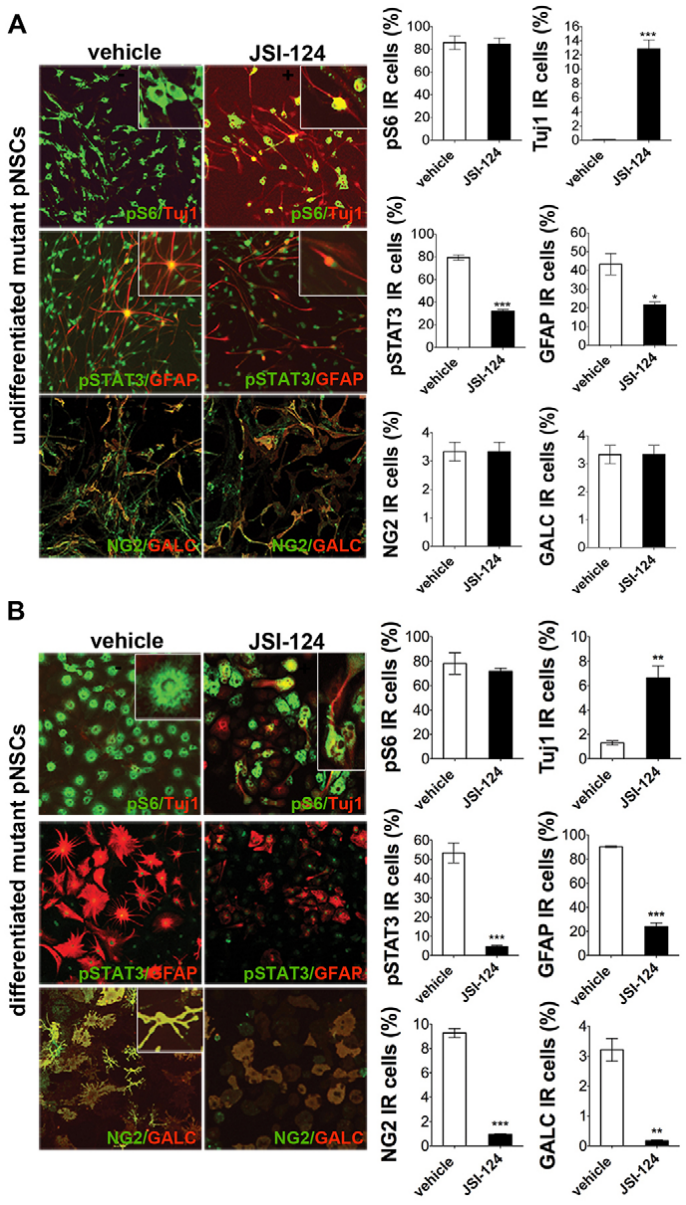
**Premature astroglial differentiation and impaired neuronal maturation of postnatal NSCs are STAT3-dependent, whereas defective oligodendrogenesis is STAT3-independent.** (A) Increased frequency of Tuj1-IR neurons and decreased numbers of GFAP-IR astrocytes in undifferentiated mutant postnatal NSC (pNSC) cultures after 72 hours of exposure to the STAT3 inhibitor JSI-124. The number of NG2/GalC-IR oligodendrocytes is not affected by treatment with JSI-124. pS6 and pSTAT3 status after JSI-124 treatment is also shown. Magnification 200×. (B) Increased frequency of Tuj1-IR neurons and decreased numbers of GFAP-IR astrocytes in mutant pNSC cultures induced to differentiate and mature by FBS addition after 72 hours of exposure to JSI-124. Again, the number of NG2/GalC-IR oligodendrocytes is not affected by treatment with JSI-124. pS6 and pSTAT3 status after JSI-124 treatment is also shown. Magnification 200×. Error bars, s.e.m. *P<0.05; **P<0.01; ***P<0.005.

To assess whether the alterations in the differentiation of mutant postnatal NSCs were also dependent on the activation of the STAT3 pathway, we exposed *Tsc1* control and mutant postnatal NSCs to the STAT3 inhibitor JSI-124 (also known as cucurbitacin) under proliferative conditions. Exposure to the inhibitor was sufficient to reduce the number of pSTAT3-IR cells and to restore the number of Tuj1-, GFAP- and NG2-IR cells in mutant postnatal NSCs to control levels (Fig. 7A), also in differentiated postnatal NSC-derived cultures (Fig. 7B). However, the reduced generation of GalC-IR oligodendrocytes in mutant postnatal NSC cultures was not efficiently rescued by STAT3 inhibition, suggesting that defective oligodendroglial maturation was STAT3-independent. Accordingly, no evidence of STAT3 hyperactivation was found in the CC of mutant postnatal brains compared with controls (not shown).

Interestingly, exposure of control postnatal NSCs to JSI-124 resulted in a dramatic decrease in the frequency of Tuj1-IR neuronal cells, suggesting that minimal activation of the STAT3 pathway is required for proper neurogenesis to take place (supplementary material Fig. S4).

## DISCUSSION

TSC is a multisystem autosomal dominant disorder, resulting from mutations in either the *TSC1* or *TSC2* gene, whose corresponding gene products, hamartin and tuberin, respectively, form a heterodimeric complex. Two thirds of TSC cases result from sporadic genetic mutations in one of the two major loci identified, i.e. *TSC1* on chromosome 9q34 and *TSC2* on 16p13. Loss of hamartin-tuberin GAP activity, as occurs in TSC-associated lesions, results in mTOR pathway hyperactivation. Because tuberin only contains the GAP-activating domain, it is commonly believed that mutations in *TSC2* give rise to a more severe phenotype than mutations in *TSC1*. However, additional pathophysiological mechanisms might be responsible for the distinct phenotypic features observed in individuals bearing mutations in either *TSC1* or *TSC2*. In fact, whereas truncating mutations in *TSC2* induce mRNA degradation and, in few cases, short non-functional proteins, non-truncating *TSC2* mutations, such as missense and small in-frame mutations, produce distinct and, in most cases, milder phenotypes than truncating mutations (van Eeghen et al., 2012; van Eeghen et al., 2013). In agreement with the high variability in phenotype, mutations in the tuberin interacting domain (TID) of TSC1 are associated with highly severe neurocognitive impairment, whereas distal mutations in the TSC2 protein, which do not affect the hamartin interacting domain (HID), result in significantly better outcomes (van Eeghen et al., 2012). As such, given the quite consistent overlap in phenotypes in individuals bearing either *TSC1* or *TSC2* mutations (Jansen et al., 2008), phenotype prediction is complicated and should be based more on the type of mutations rather than on the gene in which the mutation actually takes place (van Eeghen et al., 2012).

Recently, conditional mouse models for TSC have been generated by targeting *Tsc1* or *Tsc2* mutations in differentiated neural cells (Meikle et al., 2007; Ehninger et al., 2008; Uhlmann et al., 2002a; Zeng et al., 2011). However, none of these mouse models fully recapitulated the pathological features of the disease (Crino et al., 2006), probably as a consequence of the advanced stage of differentiation of the cells targeted by the recombination event, rather than being due to the mutation event *per se*. As such, others and we recently generated novel mouse models of TSC by deleting *Tsc1* or *Tsc2* earlier in undifferentiated embryonic progenitors (Way et al., 2009; Magri et al., 2011; Carson et al., 2012; Feliciano et al., 2011) and postnatal SVZ NSCs (Zhou et al., 2011; Feliciano et al., 2012). Notably, these conditional mouse models displayed most of the distinctive neurological features of TSC.

Besides being invaluable for their preclinical exploitability, mouse models of TSC can also be highly informative in the identification of a possible association between genotype and phenotype in TSC. A previous study exploiting astrocyte-specific (*hGFAP1*-Cre) *Tsc1* and *Tsc2* knockout models reported that, although the two models were qualitatively similar in the neurological phenotype in terms of spontaneous epilepsy, progressive glial proliferation, impaired glial buffering mechanisms and hippocampal neuronal disorganization, seizures and some histological features were more severe in *Tsc2* than in *Tsc1* mutant mice and this was due to the distinct degree of mTOR pathway activation in the two models (Zeng et al., 2011). By contrast, in the current study, we show that the radial-glia-specific (*hGFAP2*-Cre) *Tsc1* knockout mouse, in which mTOR hyperactivation was achieved through a remarkable decrease not only in hamartin but also in tuberin expression, displayed overlapping phenotypic features with the corresponding *Tsc2* knockout model (Way et al., 2009). Indeed, both mouse models, which shared the same mixed genetic background, were characterized by short lifespan, reduced weight gain, severe megalencephaly and ventricular enlargement, increased cortical thickness, cortical lamination defects, hippocampal alterations, hypomyelination, and spontaneous epilepsy. Notably, similar to the developmental defect detected in the SVZ of the NEP-specific *Tsc1* knockout model (Magri et al., 2011), an aberrant SVZ expansion was observed in RGC-specific *Tsc1* mutant mice that seemed to be present also in RGC-specific *Tsc2* mutant mice, in which it was interpreted as astrogliosis (Way et al., 2009).

Overall, the differences in the neuroanatomical and cellular pathophenotypes induced by *Tsc1* or *Tsc2* mutation in CNS-restricted mouse models, which, however, do not take into account potential differences in behavior and cognition, seem to mostly relate to the type of cell targeted by the loss of hamartin and tuberin, i.e. embryonic undifferentiated progenitors versus differentiated cells, rather than to mutation in *Tsc1* or *Tsc2*. Anyhow, given that *Tsc2* deletion in astrocytes required more than 5 weeks to induce more severe neurological disturbances than the corresponding *Tsc1* mutation (Zeng et al., 2011), we cannot rule out that the short lifespan of both RGC-targeted mutant mice might prevent the manifestation of time-dependent differences in *Tsc1*- and *Tsc2*-deletion-associated phenotypes. Inducible conditional transgenesis could be exploited to solve this issue.

Notwithstanding the many similarities detected in the two RGC-targeted models, several features remained strikingly different. As reported also in the NEP-targeted model, *Tsc1^c/−^/hGFAP2*-Cre^+^ mutant cortices did show differences in cell size only in the ectopic cortical layer detected between layer 5 and 6. Likewise, none of the many GFAP-IR cells found in the *Tsc1^c/−^/hGFAP2*-Cre^+^ mutant cortex overactivated mTOR. In addition, a significant increase in cortical thickness was observed in *Tsc1^c/−^/hGFAP2*-Cre^+^ mice as early as at E18.5, whereas, in the *Tsc2/hGFAP2*-Cre^+^ mutants, it was detected only by P10. As such, although resulting in grossly similar phenotypes, *Tsc1* mutation in RGCs leads to the development of gene-specific alterations.

Remarkably, the pivotal role of the timing of mutations in determining the severity of phenotype in TSC mouse models is supported by observations in mTOR-hyperactivating embryonic and postnatal NSCs. With respect to the NEP-targeted mouse model in which *Tsc1* loss postnatally was restricted to the dorsolateral corner of the SVZ, the *Tsc1^c/−^/hGFAP2*-Cre^+^ mutant mouse carries the relevant advantage of deleting *Tsc1* in the whole SVZ region, thus allowing the establishment of long-term expanding postnatal mutant NSC lines. In fact, studying the function of mTOR not only in the regulation of embryonic NSCs but also in postnatal NSCs is of great biological and preclinical relevance because it has been recently reported that this pathway regulates both quiescence and proliferation of SVZ progenitor pools at different stages throughout adulthood (Paliouras et al., 2012). Indeed, *Tsc1^c/−^/hGFAP2*-Cre^+^ postnatal NSCs showed an aberrant differentiation potential that was very different from the one of E16.5 *Tsc1^c/−^/hGFAP2*-Cre^+^ embryonic NSCs.

By keeping in mind that *in vitro* culturing does not fully mimic the *in vivo* microenvironment conditions, it is interesting to note that the aberrant differentiation detected in mutant postnatal NSCs can be rescued differently by mTOR and STAT3 inhibition. The defect in the differentiation of GFAP-IR astroglial cells and NG2-IR oligodendroglial progenitors was reverted by both rapamycin and JSI-124. By contrast, the decrease in GalC-IR cells *in vitro* was both rapamycin-insensitive and STAT3-independent. Remarkably, the impaired neuronal differentiation of postnatal NSCs was rapamycin-insensitive but STAT3-dependent. As such, it is tempting to speculate that different inhibitory approaches might be exploited *in vivo*, alone or in combination, in order to interfere with the distinct phenotypic features associated with TSC.

Interestingly, E16.5 mutant embryonic NSCs isolated from RGC-recombined *Tsc1^c/−^/hGFAP2*-Cre^+^ mice showed an enhanced neurogenic potential that, conversely, was lost in E16.5 mutant embryonic NSCs isolated from NEP-recombined *Tsc1^c/−^/Emx1*-Cre^+^ mice as well as in P7 postnatal NSCs isolated from RGC-recombined *Tsc1^c/−^/hGFAP2*-Cre^+^ mice. The difference in neurogenic potential might be ascribed to the time that targeted NSCs spent in the presence of strong mTORC1 hyperactivation *in vivo*. In the case of *Tsc1^c/−^/Emx1*-Cre^+^ mice, in which Cre-mediated recombination took place at E9.5, E16.5 embryonic NSCs spent 5–7 days under mTOR hyperactivation. Similarly, P7 postnatal NSCs from *Tsc1^c/−^/hGFAP2*-Cre^+^ mice, in which Cre was activated at E13.5, were exposed to mTOR hyperactivation for 10–12 days. By contrast, E16.5 embryonic NSCs from RGC-recombined *Tsc1^c/−^/hGFAP2*-Cre^+^ mice were under the activity of hyperstimulated mTOR for only 1–3 days. In further support of this notion, P7 postnatal NSC cultures isolated from the *Tsc1^c/c^*/Nestin-CreERT2^+^ mouse and Cre-recombined *ex vivo* at the same postnatal day, and, as such, not experiencing mTOR hyperactivation *in vivo*, did not display any alteration in their differentiation potential (Zhou et al., 2011). Thus, sustained activation of mTOR leads to defects in neurogenesis that are also dependent on the length of the period during which hyperactivation of mTORC1 occurs.

Overall, these findings indicate that mTOR activation in neural progenitors plays distinct roles depending on the developmental stage at which it takes place, and that genotype-phenotype correlation, at least in preclinical mouse models, might depend on the nature of the cells targeted by the mutation.

## MATERIALS AND METHODS

### Generation of *Tsc1*-floxed/*hGFAP2Cre* mice

To generate *Tsc1^c/c^hGFAP2Cre* conditional mice, mixed background (129S4/SvJae, C57BL/6) *Tsc1^c/c^* mice (Jackson) of either sex were intercrossed with male or female *hGFAP2Cre* heterozygous mice (Zhuo et al., 2001). We used ‘*c*’, ‘*wt*’ and ‘–’ to denote the conditional (floxed), wild type and null alleles of *Tsc1*, respectively; the formal name of the *c* allele is *Tsc1tm1Djk. Tsc1* mutants were genotyped by tail-derived genomic DNA PCR as described previously (Uhlmann et al., 2002b). All animal experiments were approved by and performed in accordance with the guidelines of the Institutional Animal Care and Use Committee.

### Immunostaining on paraffin-embedded and frozen sections

Brains from embryos or from intracardially perfused postnatal mice were fixed for 24 hours in 4% PFA. 2-μm paraffin and 16-μm frozen sections underwent endogenous peroxidase activity blocking by 0.3% H_2_O_2_ in methanol, were then treated for antigen retrieval and incubated with primary antibodies at room temperature (RT) for 1 hour. Primary antibodies used were: rabbit anti-pS6_S235/6_ (Cell Signaling), rabbit anti-Ki67 (Novocastra), rat anti-Ki67 (TEC3, Dako), mouse anti-NeuN (Chemicon), mouse anti-SMI311 (Covance), rabbit anti-pSTAT3_Y705_ and -pSTAT3_S727_ (Cell Signaling), goat anti-DCX (Santa Cruz Biotechnology), mouse anti-GFAP (Chemicon), rabbit anti-GFAP (Dako), rabbit anti-Iba-1 (Wako), mouse anti-MBP (Millipore), mouse anti-CNPase (Chemicon), rabbit anti-Olig2 (Millipore) and rabbit anti-S100β (Swant).

### Immunoblotting

Western blotting on cortical tissues and postnatal NSC cultures was performed as described previously (Magri et al., 2011). The primary antibodies/antisera used were: rabbit anti-pS6_S235/6_, anti-S6, anti-Tsc1 (code 4906, Cell Signaling), anti-Tsc2, anti-mTOR, anti-pmTOR_S2481_, anti-Akt, anti-pAkt_S473_, anti-ERK, anti-pERK, anti-PKCα, anti-PTEN, anti-STAT3 and anti-pSTAT3_S727_ (all antibodies from Cell Signaling), rabbit anti-pPKCα_S643_ (Millipore) and anti β-tubulin (Sigma). Reactive proteins were visualized using LiteBlot (Euroclone, Celbio) or SuperSignal West Femto chemiluminescence reagent (Pierce Biotechnology) by exposure to X-ray film (BioMax MR; Kodak).

### Video-EEG recording

Epidural stainless steel screw electrodes (0.9 mm diameter/2 mm long) were surgically implanted at P11 under sevoflurane anesthesia (Sevorane™, Abbott S.p.a. Campoverde, Italy) and secured using cyanoancrylate and dental cement (Ketac Cem, ESPE Dental AG, Seefeld, Germany). Two active electrodes were placed on right and left parietal areas (2 mm lateral to midline, 1 mm posterior to bregma) and one over the occipital area (1 mm posterior to lambda) as a common reference. After 24-hour recovery, unrestrained mice were monitored by video-EEG in recording sessions of 12–24 hours in a Faraday cage. EEG data were recorded and digitally saved using a System Plus device (Micromed, Mogliano Veneto, Italy). Tracings were filtered between 0.53 and 60 Hz. Simultaneous video data were acquired with a Canon MV550I camera connected to the recording system via Firewire. Video-EEG recordings were visually inspected off-line for detection of spontaneous seizures. Time-frequency analysis was performed offline and results were represented by density spectral arrays (DSAs).

### Isolation and culturing of NSCs

NSC cultures were established under mild hypoxic (5% O_2_) culture conditions from the embryonic cerebral cortex and the postnatal SVZ of wild-type and mutant mice. NSC long-term self-renewal was assessed as in Magri et al. (Magri et al., 2011). For clonogenic assays, cells derived from the dissociation of clonal single neurospheres were seeded in 96-well plates and the number of secondary spheres generated was assessed after 8–10 days. To evaluate multipotency, NSCs were plated onto Matrigel-coated glass coverslips in the presence of FGF2 for 3 days, and then switched to medium containing 2% FBS for an additional 4 days. Cells were fixed with 4% PFA for 20 minutes and then processed for the detection of neural antigens. Primary antibodies used were: mouse anti-GFAP (Chemicon), rabbit anti-GFAP (Dako), mouse anti-Tuj1 (Covance), rabbit anti-pS6_S235/6_ (Cell Signaling), rabbit anti-pSTAT3_S727_ (Cell Signaling), mouse anti-GalC (Millipore) and rabbit anti-NG2 (Chemicon). ToPro3 (TP3) was used for nuclear staining. The number of cells positive for each staining was normalized over the total cell number obtained by counting TP3-positive nuclei and expressed as a percentage. At least 1500 nuclei were counted for each experimental condition. Mutant postnatal NSCs were exposed to rapamycin (LC-Labs, USA) and cucurbitacin-I hydrate (JSI-124, SIGMA) at a final concentration of 100 nM.

### Treatment with rapamycin

Mice were intraperitoneally injected with 6 mg/kg of rapamycin or vehicle (5% Tween 80, 5% PEG 400) every other day.

### Statistical analysis

Results for continuous variables were expressed as mean ± standard error mean. Two-group comparisons were performed by the independent samples Student’s *t*-test. Multiple group comparisons were performed by the ANOVA test followed by Bonferroni post-hoc analysis. *P*-values <0.05 were considered statistically significant. *P<0.05; **P<0.01; ***P<0.005; ****P<0.001.

## Supplementary Material

Supplementary Material
