# Supplementary material for: Timing of mTOR activation affects tuberous sclerosis complex neuropathology in mouse models
Source: Dis Model Mech. 2013 Jun 5;6(5):1185–97. doi: 10.1242/dmm.012096 (PMC3759338; doi:10.1242/dmm.012096)
Supplement: Supplementary Material [file supp_6_5_1185__index.html]

Timing of mTOR activation affects tuberous sclerosis complex neuropathology in mouse models — Timing of mTOR activation affects tuberous sclerosis complex neuropathology in mouse models — Supplementary Material 

# Timing of mTOR activation affects tuberous sclerosis complex neuropathology in mouse models

## 

**Files in this Data Supplement:**

- **Supplementary Material PDF**
