# Supplementary material for: Timing of mTOR activation affects tuberous sclerosis complex neuropathology in mouse models
Source: Dis Model Mech. 2013 Jun 5;6(5):1185–97. doi: 10.1242/dmm.012096 (PMC3759338; doi:10.1242/dmm.012096)
Supplement: Supplementary Material [file supp_012096_DMM012096.pdf]

Supplementary Figures and Tables

Fig. S1 - Targeted inactivation of *Tsc1* in the embryonic radial glia does not result in preferential mTOR activation in selected postnatal hypothalamic nuclei and in changes in microglia activation in the postnatal cortex.

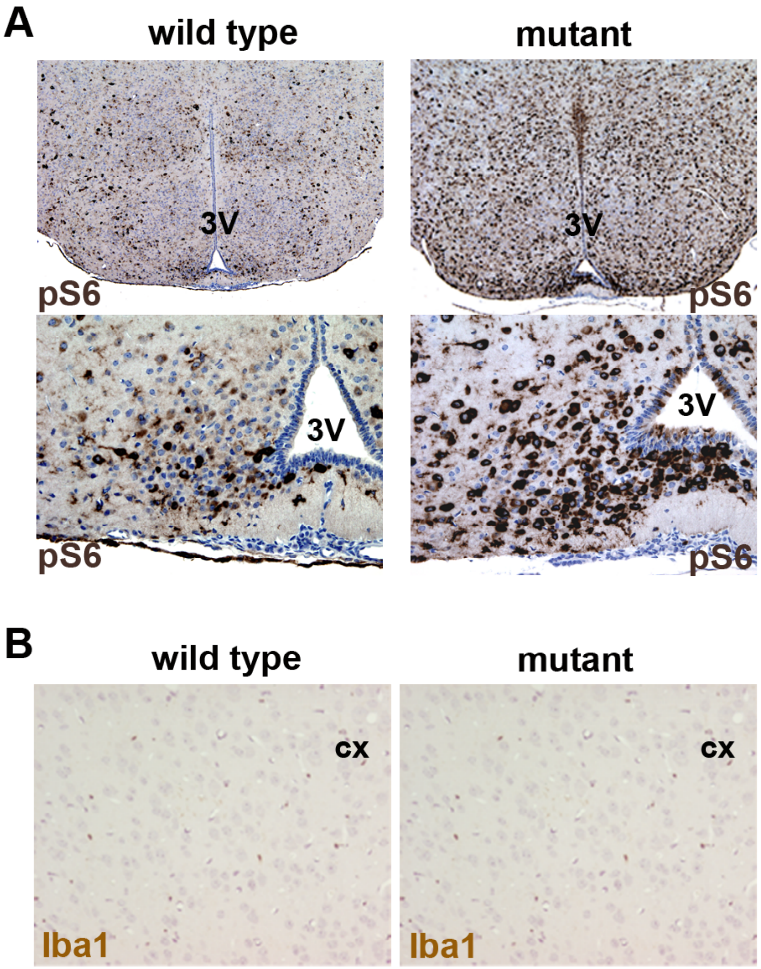

(A) hGFAP2-Cre/Tsc1$^{c/c}$ mutants displayed increased numbers of pS6-IR cells, which, however, were homogeneously distributed in the hypothalamus (3V, third ventricle; 40x; 200x). The even distribution of pS6-IR cells in the different hypothalamic nuclei did not result in significant alteration of weight in mutant mice, as opposed to the selective mTORC1 activation in the arcuate (ARC) and ventromedial hypothalamic (VMH) nuclei observed in *Emx1*-Cre/Tsc1$^{c/c}$ mice, which is known to lead to decreased food intake and poor weight gain (Magri et al, 2011).

(B) Absence of Iba1-IR microglial cells in both control and mutant cortex at P15 (cx, cortex; 200x).

Fig. S2 - Deletion of *Tsc1* in embryonic radial glia results in post-natal hippocampal alterations.

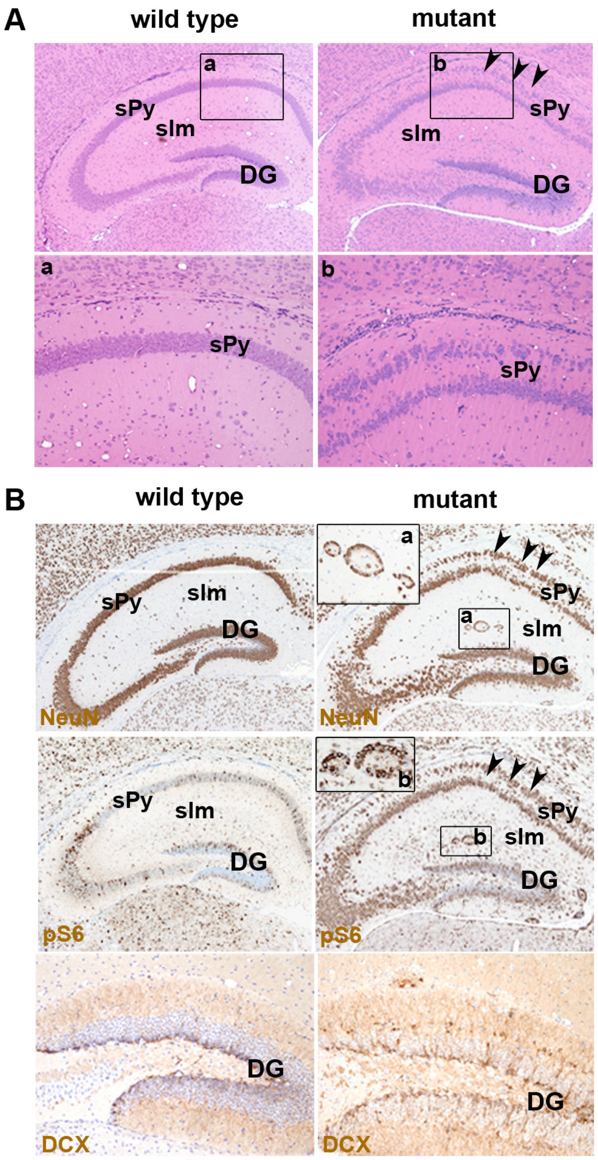

(A) Severe lamination defects in the mutant hippocampus, such as splitting of the *sPy,* are shown by H&E (arrowheads). Magnification 100x, 200x, insets 600x.

(B) Cells located in the ectopic layer of the *sPy* (arrowheads) and in ring heterotopias retrieved in the *slm* (inset *c, d*) are NeuN- and pS6-IR. *sPy:* stratum Pyramidale; *slm:* stratum lacunosum moleculare; DG: dentate gyrus; CA3: *cornu ammonis* 3; (magnification 100x, insets 600x). High pS6 activation was detected in the *sPy* in CA1/CA3 regions and in the hilus of DG in the mutant hippocampus. The mutant DG also showed increased numbers of DCX-IR neurons.

Fig. S3 - Activation of mTORC1 in embryonic (e) NSCs does not alter their differentiation into the oligodendroglial lineage.

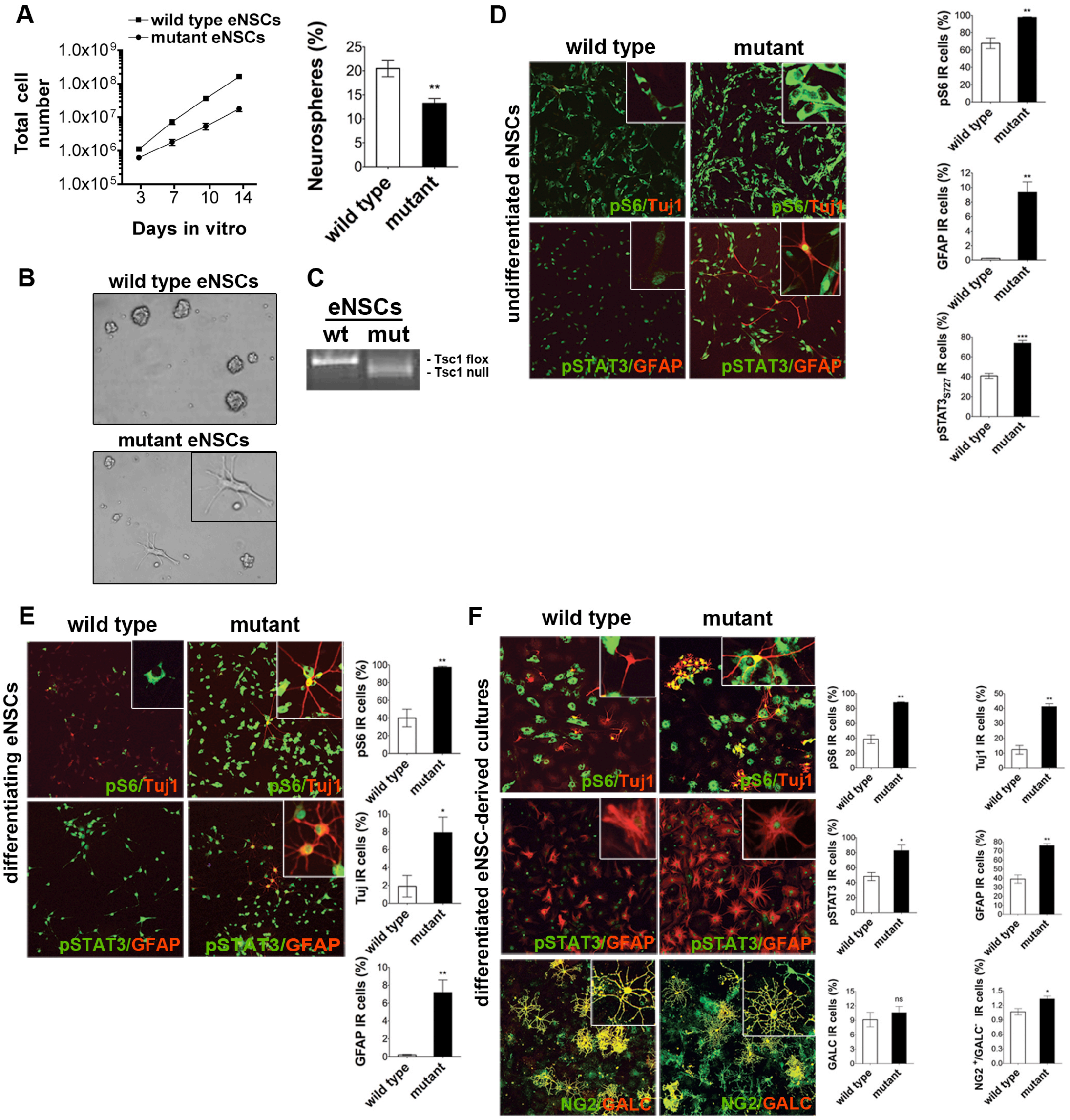

(A) Long term growth curve and clonal efficiency of control and mutant eNSCs, indicating reduced self-renewal in mutant eNSCs.

(B) Phase contrast microphotographs of control and mutant eNSCs. Magnification 200x.

(C) PCR analysis on gDNA from single mouse-derived control and mutant embryonic (e)NSCs, showing full recombination of the floxable allele.

(C) Increased pS6 phosphorylation and premature astrocytic differentiation in mutant undifferentiated eNSCs. IF showing nuclear localization of pSTAT3$_{Ser727}$ in ectopic GFAP-IR cells in undifferentiated mutant eNSC cultures. Magnification 200x.

(D) Premature differentiation into Tuj1-IR neurons and GFAP-IR astrocytes was detected in mutant eNSCs in the presence of FGF2 for 72h. pS6 and pSTAT3 immunoreactivity in differentiating eNSCs. Magnification 200x.

(E) Tuj1-IR neurons, GFAP-IR astrocytes and NG2/GalC-IR oligodendrocytes derived from control and mutant eNSCs after FBS addition. pS6 and pSTAT3 immunoreactivity in eNSC-derived differentiated cultures. Magnification 200x. Error bars, s.e.m.

Fig. S4 - Exposure of control pNSC to JSI-124 during differentiation severely impairs neuronal differentiation.

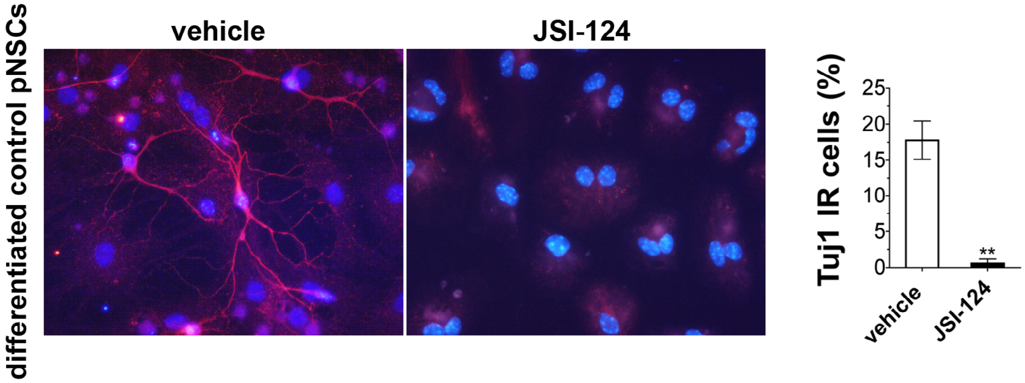

Exposure of control pNSCs to JSI-124 results in strongly diminished frequency of Tuj1-IR cells. Magnification 400x.
** $p< 0.005$, Student *t* test.

Table S1 - Video-EEG data

Recorded animals: 5 MUT, 5 WT

Hours of EEG recording in MUT mice: 287h

Hours of EEG recording in WT mice: 269h

Number of recorded seizures in MUT mice: 42

Number of recorded seizures in WT mice: 0

Seizure descriptive

Min duration [s]	Max duration [s]	Mean duration	SD duration	Seizure/h	Seizure/day
10	360	128 s	77.97 s	0.14	3.5

Individual mouse data

	Length of recording (hrs)	# Seizures	Seizure/h	Seizure/day	Mean seizure duration [s]
WT1	90	0	0	0	0
WT2	73	0	0	0	0
WT3	37	0	0	0	0
WT4	28	0	0	0	0
WT5	41	0	0	0	0
MUT1	91	10	0.11	2.64	121.6
MUT2	72	15	0.21	5.00	145.7
MUT3	47	8	0.17	4.09	113.8
MUT4	38	5	0.13	3.16	80.0
MUT5	39	4	0.10	2.46	178.8
